# The “Sorry-Bird” of Hot Springs

**Published:** 1893-09

**Authors:** 


### THE "SORRY BIRD" OF HOT SPRINGS.

There is a homely old saying that "it is a sorry bird that befouls its own nest." It is a metaphor susceptible of wide application, and we do not remember ever to have seen it so aptly illustrated as in an editorial which recently appeared in the *Hot Springs Medical Journal.*

The editorial "bird" has extensively "befouled" the medical nest, covered it all over with odium, and individually and collectively the birds of the nest are treated to a copious coat of "cussin'." If they are half as bad as they are painted by the editorial, no decent man could go in ten feet of one of them without holding his nose.

The editor says: " * * * doctors (?) are mean, and perhaps in no other profession does there lurk and skulk around more uncalled for, more childish, more cowardly hatred than in the profession that is supposed to battle in brotherly love and harmony for the general welfare of fellow man. A physician will sometimes listen to evil reports of a brother physician from the lowest source of gossip (for doctors are often thrown with the lowest classes) and will repeat it, much to the annoyance of the one maligned. When he finds what a wrong he has done by his tattling he hangs his head on meeting the injured man. This makes him feel his innate meanness, and somehow or other a bitter feeling springs up in his heart against his victim, which slowly but surely grows into hatred."

Whew! If such impression has been made upon the mind of the writer from his intercourse with and observation of the medical men of Hot Springs, he should have confined his remarks to Hot Springs. If the article were written by the senior editor, it is a most remarkable experience. He is a man who has been perhaps forty years in the practice and has always stood high. His associations, at least his opportunities for association and observation, have been with the better element of the profession and have not been confined to Hot Springs or to any one State or section, and, we say, if such has been his conviction, he must have had either a most remarkable and unfortunate experience or he has looked at things through a dense medium—a smoked glass as it were, or green glasses. That his experience has been unique we believe our readers will agree. It stands solitary and alone, unapproached by that of any one else. That it is greatly exaggerated, as the subject is seen from the standpoint of observation of the average practitioner, few will deny. For Texas, in the greater part (and we are personally acquainted in nearly every part of the State), we repudiate the assertions and the summing up. For Hot Springs, so far as our slight acquaintance with some two or three physicians whom, we always understood, were representatives of the Hot Springs profession, will warrant us in expressing an opinion, we repudiate it. Doctors do *not* "hate each other." Individual cases occur in nearly every community where jealousies exist, and perhaps some unkind words are said by one physician against another, but that "doctors hate each other," in the general application made by the editor, is demonstrably untrue. There is more of the milk of human kindness, more true charity in the well-bred physician than in any other class on earth.

The writer says: "A physician will sometimes listen to evil reports of a brother physician from the lowest source of gossip (for doctors are often thrown with the lowest classes) and will repeat it." That is a severe accusation and will not be endorsed, we believe, by any one who has had intercourse with the better element of the profession. No one worthy of the name "physician" would be guilty of such conduct; and for those calling themselves "physician," of whom that charge can be justly made, a good horse whipping is clearly indicated and ought to be administered. It would be hard to think of certain high minded and honorable men in the profession at Hot Springs in the same breath with such a charge. True, physicians *are* often "thrown with the lowest classes," but we have always understood that though his calling made it necessary to go amongst and minister to the sick amongst the vicious and depraved, he never descended necessarily to their level, or had aught in sympathy with them, except as to their suffering. The physician has also to go amongst filth, to put his hands into a gangrenous sore, but he is not thereby contaminated.

Our experience in several sections of country, in several states has been that, *as a class*, there is more friendship, charity and brotherly love, more disposition to screen from criticism a weak brother and to help hlm, than in most other professions. That social intercourse amongst them and their families has been pleasant and agreeable.

The *New York Medical Record*, commenting on this same editorial, says: "The measure of professional advance is pretty accurately determined by the amount of local quarreling. In proportion as the standard of mental and medical attainment increases, the small jealousies disappear." A sentiment we endorse. All is not gold that glitters. Every smooth-tongued and well-dressed scamp that calls himselt "doctor" is not a physician, and we learn that Hot Springs is full of that sort; but the editor of the *Hot Springs Medical Journal* is not speaking alone of Hot Springs and makes no exceptions. His digestion must be ont of gear. He is "bilious," and having a bad taste in his mouth he wants everybody else to have it. We repudiate his assertions and conscientiously defend the "nest" against such attempts to befoul it.

